# STARD3: A Prospective Target for Cancer Therapy

**DOI:** 10.3390/cancers13184693

**Published:** 2021-09-18

**Authors:** Kanwal Asif, Lorenzo Memeo, Stefano Palazzolo, Yahima Frión-Herrera, Salvatore Parisi, Isabella Caligiuri, Vincenzo Canzonieri, Carlotta Granchi, Tiziano Tuccinardi, Flavio Rizzolio

**Affiliations:** 1 Department of Molecular Sciences and Nanosystems, PhD School in Science and Technology of Bio and Nanomaterials, Ca’ Foscari University of Venice, 30172 Venice, Italy; 956538@stud.unive.it; 2 Pathology Unit, Centro di Riferimento Oncologico di Aviano (C.R.O.) IRCCS, 33081 Aviano, Italy; spalazzolo@cro.it (S.P.); salvoparisi94@gmail.com (S.P.); vcanzonieri@cro.it (V.C.); 3 Department of Experimental Oncology, Mediterranean Institute of Oncology, 95029 Catania, Italy; lorenzo.memeo@grupposamed.com; 4 Department of Molecular Sciences and Nanosystems, Ca’ Foscari University of Venice, 30172 Venice, Italy; yahima.frionherrera@unive.it or; 5 Department of Medical, Surgical and Health Sciences, University of Trieste, 34149 Trieste, Italy; 6 Department of Pharmacy, University of Pisa, 56126 Pisa, Italy; carlotta.granchi@unipi.it (C.G.); tiziano.tuccinardi@unipi.it (T.T.)

**Keywords:** STARD3, targeted drugs, cholesterol, steroidogenic acute regulatory transfer proteins, inhibitors, cancer

## Abstract

**Simple Summary:**

Alterations in cholesterol level play an important role in cancer development. Lipid transfer proteins (LTPs) are involved in cholesterol distribution between organelles. Among LTPs, some members of steroidogenic acute regulatory-related lipid transfer (START) protein family regulate the cholesterol transportation between organelles and have been revealed as critical for cancer development. This review highlights the recent discoveries of the StAR-related lipid transfer protein domain 3 (STARD3) member of START proteins in cancer development and progression. Blocking cholesterol transportation through the inhibition of STARD3 activity could be an important strategy to treat cancer.

**Abstract:**

Cancer is one of the major causes of death in developed countries and current therapies are based on surgery, chemotherapeutic agents, and radiation. To overcome side effects induced by chemo- and radiotherapy, in recent decades, targeted therapies have been proposed in second and even first lines. Targeted drugs act on the essential pathways involved in tumor induction, progression, and metastasis, basically all the hallmark of cancers. Among emerging pathways, the cholesterol metabolic pathway is a strong candidate for this purpose. Cancer cells have an accelerated metabolic rate and require a continuous supply of cholesterol for cell division and membrane renewal. Steroidogenic acute regulatory related lipid transfer (START) proteins are a family of proteins involved in the transfer of lipids and some of them are important in non-vesicular cholesterol transportation within the cell. The alteration of their expression levels is implicated in several diseases, including cancers. In this review, we report the latest discoveries on StAR-related lipid transfer protein domain 3 (STARD3), a member of the START family, which has a potential role in cancer, focusing on the structural and biochemical characteristics and mechanisms that regulate its activity. The role of the STARD3 protein as a molecular target for the development of cancer therapies is also discussed. As STARD3 is a key protein in the cholesterol movement in cancer cells, it is of interest to identify inhibitors able to block its activity.

## 1. Introduction

Cancer, one of the leading causes of death worldwide [1], is a complex disease in which the number of cells increases uncontrollably. Alterations in the DNA and different risk factors, such as physical inactivity, smoking, unbalanced diet, and the environment are involved in this process [2]. The World Health Organization (WHO) has estimated that the number of cancer patients and related deaths could increase rapidly with the growth and aging of the population or as a result of unhealthy lifestyles [3,4,5]. The highest number of cases and deaths are from breast, lung, and colorectal cancers in women and prostate, lung, and colorectal cancers in men. However, the incidence also continues to increase for other cancers such as the pancreas, kidney, stomach, liver, oral cavity, and melanoma of the skin in both sexes [1,6].

To reduce cancer mortality, a better biological understanding of these malignancies could lead to the development of more specifics and effective drugs prolonging the quality of life and the survival of cancer patients [7]. Following this idea, researchers are focusing on the identification of different proteins for the successful development of molecular-targeted therapies that can block novel signaling pathways [8,9,10,11,12,13]. In recent years, various molecular-targeted agents have been approved by the US Food and Drug Administration (FDA), including small molecules and antibodies [12] used to target the proteasome [14], cyclin-dependent kinases (CDKs) [15], epidermal growth factor receptor (EGFR) [16], vascular endothelial growth factor (VEGF) [17], poly (ADP-ribose) polymerase (PARP) [18,19], and programmed death-1 (PD-1)/programmed death-ligand 1 (PD-L1) [20]. Among novel molecular targets, cholesterol metabolism has received increasing attention due to its role in cancer development [21,22]. Recent studies have suggested that cancer cells reprogram the cholesterol metabolism to regulate the permeability and fluidity of the cell membrane and increase the transduction of intracellular survival signals [23,24,25]. Zhuang et al. provided evidence that cholesterol, through the formation of lipid rafts, is a mediator of signal transduction processes relevant to prostate cancer cell survival and disease progression both in vivo and in vitro [26].

**Figure 1:**
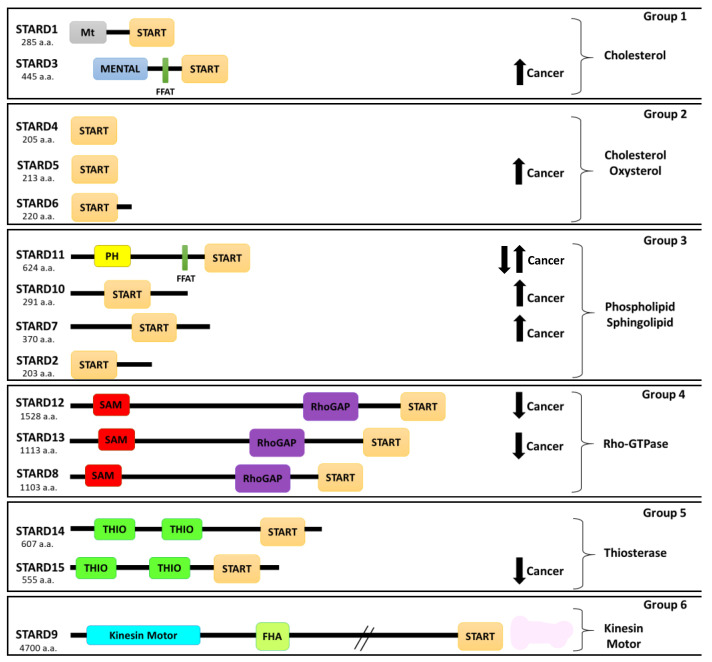
Schematic representation of the START proteins grouped into six subfamilies. START (StAR-related lipid-transfer domain), Mt (mitochondrial targeting sequence), MENTAL (MLN64 NH(2)-terminal domain), PH (pleckstrin homology domain), FFAT (two phenylalanines in an acidic tract), RhoGAP (Rho-GTPase-activating domain), SAM (sterile alpha motif domain), THIO (thioesterase active domain), and FHA (N-terminal Forkhead-associated domain). a.a.—amino acids. Arrows indicate overexpression (up) or downregulation (down) of protein in cancer.

Intracellular cholesterol and its synthesis are mediated by a complex protein network, including sterol regulatory element-binding proteins (SREBPs) [27,28,29], SREBP cleavage-activating protein (SCAP) [28,29,30], 3-hydroxy-3-methylglutaryl coenzyme A reductase (HMG-CoA reductase) [29], insulin induced-genes (Insigs) [28,31], cytosolic sterol carrier protein 2 (SCP2), fatty acid-binding protein (FABP) transfer sterols [29,32], oxysterol binding proteins (OSBPs) [33], protein aster-A (GRAMD1) [34], late endosomal oxysterol-binding protein homologue (ORP1L) [35]; and Niemann Pick type-C1 protein (NPC1), Niemann–Pick type-C2 protein (NPC2) [36], and Steroidogenic acute regulatory (StAR) related lipid transfer proteins (START) [29,37]. These proteins are involved in the vesicular traffic of cholesterol through the endocytic and secretory pathways as well as in non-vesicular exchange between organelles [29,37]. Currently, non-vesicular cholesterol transport has gained great interest in the scientific community due to the possible association between lipid transfer proteins (LTP) and cancer development [29]. START proteins are a family of LTPs involved in different cellular processes, including non-vesicular cholesterol transport [38]. The START family is composed of 15 members grouped into six subfamilies according to the similarity of the amino acid sequence and ligand binding (Figure 1) [38,39]. There are published data that suggest that StAR-related lipid transfer domain-3 (STARD3), a membrane-targeted START protein, regulates the cholesterol accumulation in endosomes and mediates its inter-organelle distribution [40,41,42]. STARD3 is a member of a subfamily of lipid trafficking proteins that are characterized by a C-terminal START domain, a central FFAT domain, and an N-terminal MENTAL domain. Different studies have reported the association of STARD3 expression with several cancer types [43,44,45,46], and in breast cancer patients, a high level of STARD3 is associated with metastasis, local recurrence, and shorter overall survival [47,48]. Differently from others START proteins, STARD3 is mapped in 17q12-21, a chromosomal region frequently amplified in many cancers including breast, colorectal, and gastric cancers [49,50,51]. For these reasons, STARD3 could be considered a potential oncogene for which the first inhibitor was reported [52].

To the best of our knowledge, for the first time, we summarize the role of STARD3 in cancer and computational strategies to propose possible binding sites of potential STARD3 inhibitors and challenges for the discovery of new inhibitors.

## 2. The Role of Intracellular Cholesterol in Cancer Development

Several studies indicated the association between cholesterol levels and cancer development [21]. In fact, serum cholesterol is associated with an increased risk of more than 10% in prostate cancer recurrence [53,54]. Other studies also suggest its association with an increased risk of colorectal [55,56], lung [57,58], and breast cancers (BC) [59,60]. The use of statins, a class of lipid-lowering medications, is associated with a reduction risk of melanoma, non-Hodgkin lymphoma, and endometrial and breast cancers [61,62,63]. The effect of statins after colorectal cancer diagnosis is described in another report highlighting a dose-dependent reduction in colorectal cancer mortality linked with statin administration [64]. On the other hand, epidemiological studies demonstrated contrasting results between serum cholesterol and cancer; in some cases, it was found that low level of cholesterol and statins are associated with oncogenic effects [65]. These data suggest that the alteration of one of the multiple mechanisms that regulate cholesterol homeostasis could promote cancer development.

Recent studies have shown that intracellular cholesterol levels in cancer cells may be more important than serum cholesterol levels [66]. Moreover, intracellular cholesterol homeostasis varies between different cancer types, and consequently, its cellular activity change [67]. Therefore, intracellular cholesterol level seems to be more important than dietary cholesterol level in cancer development. Cholesterol is an essential component of mammalian cells and comprises approximately 30% of the plasma membrane [68]. This type of structural lipid is synthesized in the liver and transported by low-density lipoprotein (LDL) to target cells throughout the body [69]. The intracellular _cholesterol_ uptake is mediated by membrane receptors, including LDL receptors, several LDL receptor-related proteins (LRP1, LPR2, LRP5, LRP6, and LPR8), and others [70]. Inside the cells, the distribution of cholesterol occurs through vesicular and non-vesicular transport mechanisms [71,72]. Indeed, there are differences in the cholesterol content between the membranes of cell organelles, which are partially attributed to the interaction of cholesterol with membrane phospholipids [71]. Interestingly, non-vesicular transport plays an important role in maintaining the correct distribution of cholesterol between organelles [72], and several proteins, such as the START family, are involved, as described below [38,73,74,75].

Published data indicated that cancer cells showed higher levels of intracellular cholesterol compared to nontumor cells [76,77,78]. Cancer cells require high amounts of nucleic acids, proteins, and lipids for their survival [79]. Thus, to cope with these high requirements, cancer cells modify their metabolism. Among metabolic changes, cancer cells increase the de novo lipid biosynthesis including cholesterol synthesis [80,81,82]. It has been reported that several cancer cell types have higher membrane cholesterol levels and are richer in lipid rafts [77]. Cholesterol accumulation participates in the formation of sphingolipid-rich membrane microdomains and stimulates the progression and migration of cancer cells [23,24,25]. Human breast (MDA-MB-231 and MCF-7) and prostate cancer (LNCaP and PC-3) cell lines had a higher cholesterol level compared to their nontumorigenic counterparts [77]. It was demonstrated that a decrease in cholesterol from plasma membrane is related to anoikis-like apoptosis and the progressive increases in membrane cholesterol could contribute to the expansion of rafts/caveolae in order to potentiate oncogenic pathways [77]. Badana et al. showed that methyl-β-cyclodextrin, a cholesterol depleting agent, has an effect on lipid rafts of breast cancer cell lines (MDA-MB468 and MDA-MB231), decreases cell proliferation and migration, and induces apoptosis [83].

Likewise, Raghu et al. demonstrated that lipid raft disruption in several breast cancer cell lines caused a decrease in migration and cell invasion compared with nontumor cells [84,85].

Intracellular cholesterol and lipid rafts are associated with an increasing number of oncogenic signals and proteins involved in cancer progression, cell invasion, and metastasis. It has been demonstrated that AKT/mTORC1/SREBP pathway (Protein kinase B/mammalian target of rapamycin complex 1/sterol and regulatory element-binding protein pathway) induces the synthesis of cholesterol and contributes to tumor cell growth [21,86]. In prostate cancer, upregulation of the intracellular cholesterol levels mediated by the AKT pathway promoted cancer aggressiveness and bone metastases [87,88]. Additionally, the activation of aberrant p53-mediated cholesterol synthesis induces the proliferation of breast cancer cells via prenylation of Rho GTPase proteins [66]. An increase in the expression of some START proteins has also been associated with cancer cell proliferation, metastases, and resistance to chemotherapy [21,38,44,89].

## 3. Role of Protein-Related Lipid Transfer (START)/Domain (STARD) Family in Cancer

In humans, there are 15 members of START domain proteins, which are subdivided into six groups according to their amino acid sequence similarity and lipid ligands [90,91]. Groups 1 and 2 consist of cholesterol-binding proteins (STARD1/D3 and STARD4/D5/D6, respectively); group 3 binds multiple ligands, such as phosphatidylcholine (STARD2, (phosphatidylcholine transfer protein) PCTP/D7/D10) and ceramides (STARD11); group 4 proteins contain the Rho-GTPase-activating domain (STARD8/D12/D13); group 5 contains two thioesterase active domains (STARD14/D15); while group 6 consists of only a single member STARD9 with a motor domain and an FHA domain (N-terminal Forkhead associated domain) (Figure 1) [91,92].

Alterations of START proteins may cause several pathological conditions [89], including cancer, by modifying critical signaling pathways (Table 1).

STARD4 was found to be overexpressed in breast cancer and associated with distant metastasis-free survival time. Knockdown experiments have shown that STARD4 induces cell proliferation and migration and decreases apoptosis [96].

STARD5 is a cytosolic protein involved in sterol transport, expressed predominantly in liver Kupfer cells and in kidney [97,123]. It was observed that STARD5 is able to bind not only to cholesterol but also 25-hydroxycholesterol, a potent mediator of inflammatory processes and regulatory oxysterol [123]. The function of STARD5 in cancer development is unclear. However, STARD5 induction upon endoplasmic reticulum (ER) stress suggests a role of this protein in the development of neoplasia through alterations of the intracellular cholesterol distribution, implying modifications in membrane fluidity and permeability [124]. Sharon et al. described that the expression of STARD5 is significantly increased in about 70% of human colon cancer tissue compared with normal controls, highlighting that this protein could be involved in colon cancer proliferation [98].

STARD7 is a phosphatidylcholine (PC) transfer protein that mediates the uptake of PC by the mitochondria [125]. Several RT-PCR analyses in different tumor cell lines showed that STARD7 had a high expression in human-trophoblast-derived cells (HTR8-SVneo, JAR, and JEG-3), colorectal adenocarcinoma (HT29 and Caco-2 cells), and hepatocellular carcinoma HepG2 cells [126]. In contrast, low expression of this protein was found in human breast adenocarcinoma MCF7 cells, human cervix adenocarcinoma HeLa cells, human melanoma SK-MEL-31 cells, human lung adenocarcinoma A549 cells, human promyelocytic leukemia HL-60 cells, and human acute myelocytic leukemia K-562 cells [99,126].

STARD9 is localized in the centrioles. Torres et al. indicated that this protein has an important role in the integrity of the pericentriolar materials and could be a trigger for apoptosis in STARD9-deficient cells. Although the role of STARD9 in cancer development is still unknown, the inhibition of STARD9 function may be an appropriate approach to inhibit the division of cancer cells [127,128].

Several works suggest the role of STARD10 in deregulating cell growth and tumorigenesis. This protein is highly expressed in approximately 60% of human breast cancer cell lines, in 30% of primary breast carcinomas, and in mouse mammary tumors [110]. STARD10 was found to be coexpressed with ERBB2 in certain breast carcinoma cell lines, suggesting an increase in cell proliferation for tumors expressing both proteins [110,129].

STARD11/CERT belongs to the phospholipid/ceramide subgroup of START family and is characterized by pleckstrin homology domain (PH), a middle region of two phenylalanines in an acidic tract (FFAT), and a START domain in the C-term. Its physiological role has been revealed by Hanada et al.—thanks to the PH domain, it is bound to Golgi apparatus while the FFAT motive interacts with ER by VAP proteins, leaving the START domain free to mediate trafficking of ceramide at membrane contact sites between the ER and the Golgi [130]. STARD11’s role in cancer is controversial. Immunohistochemistry analysis on breast cancer tissue samples has revealed that STARD11 is downregulated in the basal-like BC subtype [113] but, conversely, CERT expression is overexpressed in HER2-positive BC and associated with poor prognoses [114]. Moreover, data analysis on clinical trials have associated STARD11 levels expression to paclitaxel resistance in BC [131] and ovarian cancer [132].

Conversely, published data have shown that Rho GTPase-activating proteins (STARD12/D13) inhibited cancer cell growth. Numerous studies have indicated that STARD12 inhibited the cell growth, invasiveness, and tumorigenicity of human breast, ovarian, liver, nasopharyngeal, and non-small-cell lung cancer cells [117,133,134,135]. Likewise, STARD13 has been shown to inhibit the proliferation of liver and breast cancer cells and, moreover, suppress the Ras-induced transformation of rodent cells [136,137]. STARD15 has also been considered a target to inhibit neoplastic cell growth. This protein has been linked to cholesterol metabolism, as its activity increases when cholesterol synthesis is inhibited [91].

In summary, the expression and activity of several START proteins may be involved in tumor development and metastasis. Here, we will focus on STARD3 and discuss its role in cancer development.

## 4. Structural Analysis of STARD3

The human STARD3 protein is composed of 445 residues. The first 170 residues correspond to the transmembrane portion that comprises four helical regions of approximately 20 residues [138]. Unfortunately, there is no 3D information in the literature concerning this protein region, nor is it possible to construct a homology model [139,140]. The only possibility to obtain a 3D model of this protein region would be the development of an ab initio model [141].

**Figure 2:**
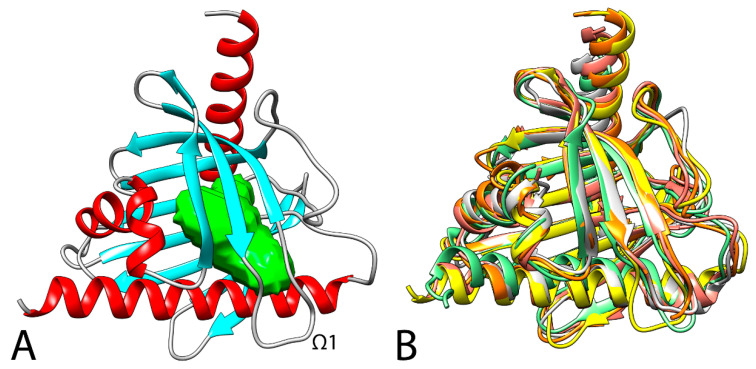
(**A**) 3D structure of STARD3 (5I9J). The cavity of the cholesterol binding pocket is colored in green. (**B**) Superimposition between the secondary structure of STARD1 (3P0L, orange [145]), STARD3 (5I9J, yellow [146]), STARD4 (6L1D, white [147]), STARD5 (2R55, salmon [148]), and STARD6 (2MOU, green [149]). RSMD values for the alignments compared with STARD3: STARD1, 1.3 Å; STARD4, 2.8 Å; STARD5, 2.7 Å; STARD6, 3.0 Å.

The domain that distinguishes STARD3 from the other START domain proteins is called MENTAL (MLN64 NH2-terminal). This domain anchors the protein to late endosomal membranes, exposes the START domain in the cytosol, and mediates homotypic as well as heterotypic interactions between STARD3 and its paralog, the STARD3 N-terminal like (STARD3NL) protein [42]. This domain interacts with cholesterol in vivo, supporting the possibility that it acts as a sterol reservoir [91,142]. The protein region corresponding to the 170–445 sequence constitutes the cytoplasmatic domain of the protein, which is responsible for binding to cholesterol. Two crystal structures of the apo START domain, at 2.2 Å and 1.74 Å resolution, were published (1EM2 [143] and 5I9J [144] PDB code, respectively). As shown in Figure 2, the domain adopts a helix-grip fold with a nine-stranded β-sheet and three α-helices, with the cavity entrance guarded by an omega loop (Ω1) that connects the β5 and β6 strands.

An analysis with BLAST software [150] indicated that among the other human proteins, beyond its close paralogue STARD3NL (STARD3 N-terminal like), STARD1 is the one with the highest similarity, is smaller than STARD3, and shows 31.6% of conserved residues with respect to STARD3. In addition, the cholesterol molecule located within the STARD1–START binding pocket has a lower degree of freedom than the cholesterol molecule within the STARD3–START tunnel, indicating differences in the orientation of the cholesterol ring within the cavities of each protein [151]. The other STAR proteins show a lower level of similarity; however, as shown in Figure 2, a superimposition between the available human STARD1, STARD3, STARD4, STARD5, and STARD6 x-ray structures highlights the same secondary structure. In STARD3 and STARD1, the conserved Arg351 (STARD3), an acidic residue at position 332 (Asp in STARD3 and Glu169 in STARD1), and the conserved Gln421 (position 258 in STARD1) are identical or highly conserved residues, which could be important for ligand binding. Interestingly, the electron-density maps reported by Horvath and coworkers for STARD3 (5I9J PDB code [146]) highlight two alternative conformations for Arg351—one of which is able to form a salt-bridge with Asp332 that partially blocks the internal cavity of the protein and could act as an allosteric trigger point in the communication with retinal proteins and enzymes involved with xanthophyll transport and metabolism [144].

## 5. Binding Mode of Cholesterol with STARD3

Photoaffinity labelling studies suggested that the cholesterol molecule located within the STARD1–START binding pocket has a lower degree of freedom than the cholesterol molecule within the STARD3–START tunnel, indicating differences in the orientation of the cholesterol ring within the cavities of each protein [151]. The x-ray structures reported in the literature represent an important tool for the analysis of STARD3 but cannot clearly shed light on the orientation of cholesterol as no x-ray complexes between STARD3 and cholesterol have been reported in literature until now. Cavity analysis of the x-ray structure revealed the presence of a single potential binding site for cholesterol. As shown in Figure 2, this cavity is delimited by seven β-sheets and two of the three α-helices. In 2000, Tsujishita et al. hypothesized that the 3-hydroxyl group of cholesterol could interact with Arg351, and the rest of the molecule could make extensive hydrophobic contacts with the other residues of the cavity [143].

In order to provide a possible orientation of cholesterol inside STARD3, in 2006, Murcia et al. [152] generated an ensemble of 100 protein conformations by means of a 1-ns molecular dynamics (MD) simulation at 500 K, in which the protein’s backbone remained harmonically restrained. After the docking of cholesterol, the 20 best-scoring solutions for each protein conformation were further analyzed.

The obtained 2000 docking poses were clustered and, from the resulting four subfamilies, the analysis of the binding energies for each of the ensembles generated with MD simulations suggested two potential cholesterol orientations (defined as IN-1 and OUT-2), with the IN-1 as the most probable.

Following this last binding hypothesis, cholesterol forms a hydrogen bond at the end of the cavity with both the side chain and the backbone carbonyl of S362, and a series of hydrophobic contacts [152]. In 2018, Kumar et al. reported a hypothetical binding orientation of cholesterol into STARD3 obtained by means of an ensemble docking analysis. Based on this approach, the ligand shows significant lipophilic interactions with Val314, Trp404, Leu406, Leu410, Leu423, and Thr426. No H-bonds between the ligand and the protein were observed [153].

In 2019, Lapillo et al. reported a third hypothesis regarding the interaction of cholesterol inside STARD3 [43] by applying a consensus docking (CD) strategy [154] followed by MD simulations and Molecular Mechanics Poisson–Boltzmann Surface Area (MM-PBSA) calculations [155]. Following this approach, the phenanthrene core of cholesterol forms hydrophobic interactions with V314, F388, W404, and L406, whereas the terminal aliphatic chain interacts with A337, F347, and L410. Finally, in agreement with Murcia and coworkers, the hydroxyl group of cholesterol forms an H-bond with the hydroxyl group of S362.

## 6. Role of STARD3 in Human Cancers

STARD3 is a transmembrane protein involved in cholesterol transfer and localized in late endosomes (LE). STARD3 can also induce the movement of lysosomal cholesterol into the mitochondria, stimulating steroidogenesis [156,157,158,159]. Several reports indicated that elevated levels of mitochondrial cholesterol could inhibit apoptotic cell death in different cancer types—in turn, inducing tumor progression [44,160]. STARD3 is mapped in 17q12-21 close to the amplicon of the HER2 gene. It was demonstrated that several of these genes, including STARD3, could functionally contribute to the proliferation of cancer cells that present amplification of HER2 [161]. The amplification/overexpression of STARD3 in cancer potentially could stimulate an independent steroidogenesis helping the promotion of hormone-driven cancers, such as breast and prostate cancers [45,46,47]. Vassilev et al. found an increased level of STARD3 in 10% of breast cancer patients associated with HER2 amplification, high Src activity, and poor patient survival [44].

Indeed, coamplification of STARD3 and HER2/receptor tyrosine-protein kinase erbB-2 (ERBB2) genes contributes to the proliferation and metastasis of breast cancer cells by increasing membrane cholesterol and thereby improving oncogenic signaling [44,161,162,163]. Differently, downregulation of STARD3 has been demonstrated in triple-negative breast cancer [164].

Yun et al. highlighted that a PPP1R1B–STARD3 fusion transcript was found to be overexpressed in 21.7% of primary human gastric cancers but not in adjacent matched normal gastric tissues [165]. In vitro experiments on MKN-28 cells demonstrated that the overexpression of PPP1R1B–STARD3 significantly increased cell proliferation and colony formation. This increased proliferation was mediated through the activation of phosphatidylinositol-3-kinase (PI3K)/AKT signaling [165]). Furthermore, in in vivo experiments on athymic nude mice bearing MKN-28 tumors, PPP1R1B–STARD3 enhanced tumor growth. In a study by Qiu et al., the authors found a higher expression of STARD3 in tubular and papillary adenocarcinoma compared with poorly differentiated adenocarcinoma cells. It was speculated that this data could be related to the abundance of mitochondria in tubular and papillary adenocarcinoma cells [50].

In prostate cancer, it was found a linear correlation between the expression of STARD3 and CYP17, an enzyme involved in the steroid biosynthesis pathway [166]. In addition, the authors thought that STARD3 and CYP17 expression in prostate cancer could lead to steroidogenesis through continuous cholesterol transfer into the mitochondria, increasing androgen biosynthesis via the catalytic activity of cytochrome CYP17. In this regard, unbalanced expression of STARD3 and CYP17 is associated with a poor prognosis in prostate cancer patients [46].

**Figure 3:**
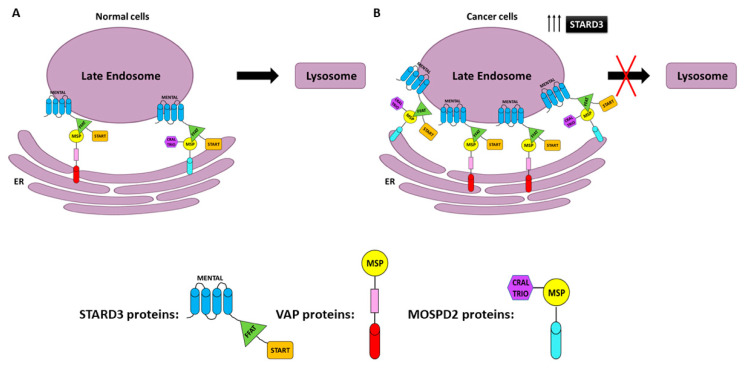
Endoplasmic reticulum–endosome interaction in normal (ER–LE) (**A**) and cancer cells overexpressing STARD3 (**B**). The STARD3/START domain interacts with vesicle-associated membrane-protein-associated proteins (VAP) or motile sperm domain-containing protein 2 (MOSPD2) to form ER–LE. FFAT (two phenylalanines in an acidic tract), MENTAL (MLN64 NH (2)-terminal domain), MSP (major sperm protein domain), and CRAL-TRIO (amino-terminal domain).

Although the molecular mechanism is still unclear, these data show that a high expression of STARD3 influences the accumulation of membrane cholesterol, which could contribute to cancer aggressiveness. Some studies have demonstrated its role in cholesterol shuttling between ER and endosomes [167]. The integral ER membrane proteins vesicle-associated membrane-protein (VAMP)-associated protein (VAP) is a protein family (VAP-A and VAP-B) found in almost all eukaryotes, which interact with STARD3 [42,44,167,168]. STARD3 has a nonconventional FFAT motif (it contains two nonconventional phenylalanines in an acidic tract (FFAT) protein motif) with seven core residues in an acidic flanking region that contains a serine residue at the 4th position instead of an acidic residue and is able to bind VAP proteins and MOSPD2 (motile sperm domain-containing protein 2) through the interaction with the MSP domain [169] (Figure 3). It was supposed that abnormal ER structures called karmellae could be produced by ER–endosome interaction in response to overexpression of STARD3, which can block the LE and inhibit its maturation to lysosomes (Figure 3B) [167,170,171,172].

Under this state, lysosomal degradation activity could be compromised. Cell surface growth factors receptors, such as HER2, may be not degraded, leading to uncontrolled cell growth as a consequence of a continuing signal transduction [149]. Along these lines, STARD3 may increase the progression of HER2-positive cancer. This hypothesis is supported by the experimental data obtained by Vassilev and colleagues, who showed that STARD3 overexpression enhances oncogenic signals in breast cancer cell lines [45].

**Figure 4:**
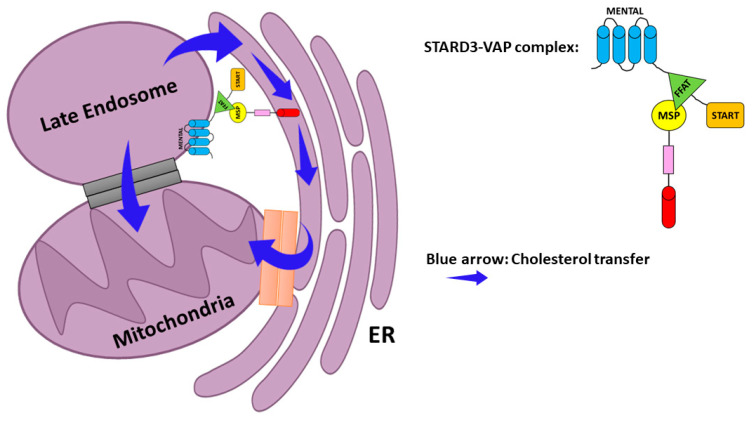
Possible model for cholesterol transport to mitochondria. ER–Late endosome interaction via the STARD3–VAP complex. Cholesterol is sorted to the mitochondria via either the ER–mitochondria MCS or MAM (mitochondria-associated ER membrane) (orange). Contact between endosome membranes and the mitochondrial membrane also allows direct transfer of cholesterol (Gray). Blue arrow—cholesterol transfer.

Another possible molecular mechanism played by STARD3 during tumorigenesis is through mitochondrial intermembrane trafficking of cholesterol [21,160]. Although mitochondria have a low content of cholesterol compared with other organelles, as mentioned above, cancer cells have higher levels of mitochondrial cholesterol that could constrain cell death by inhibiting the release of apoptotic proteins from mitochondria. Some researchers hypothesized that STARD3 could be involved in cholesterol transport from late endosomes to the ER and subsequently to the mitochondria via the mitochondria-associated ER membrane (MAM) [167,173,174]. The authors indicated that the STARD3–VAP complex could induce the formation of membrane contact sites (MCS) [174] (Figure 4). Although the STARD3–MOSPD2 complex does not participate in the formation of ER–mitochondria MCS [175], the association of the ER with both late endosomes and mitochondria through MCSs scaffolded by VAPs may contribute to cholesterol transport [167,174]. It was demonstrated that STARD3 depletion dramatically reduced lysosome–mitochondria MCS in NPC1-deficient cells, indicating the crucial role of STARD3 in the MCS formation and the regulation of cholesterol transport to the mitochondria [158,176].

As STARD3 is a cholesterol-specific START protein, the challenge is to switch off the abnormal function or expression of this protein in cancer cells. In line with this data, STARD3 could be a molecular target for therapeutic treatments pointed to the lipid metabolism of neoplastic cells.

### STARD3 Somatic Mutations in Cancer

An analysis of the COSMIC (Catalog of Somatic Mutations in Cancer) database reveals a number of STARD3 somatic mutations in several tumor types. COSMIC is an expert-curated database of somatic mutations reported in the scientific literature or from the Cancer Genome Project, exploring the impact of somatic mutations in human cancer [177]. The data from COSMIC refers to 38,259 unique samples from patients with different types of cancer including breast, lung, liver, ovary, skin, nervous system, intestinal, and stomach cancer (retrieved on 26 April 2021). The analysis was conducted applying the following filter/criteria: it was considered only mutations present in tumor samples (not cultured samples) where both tumor samples and germ-line samples of the same patient have been analyzed and the variant allele is present only in the tumor sample (confirmed somatic mutation); or no germ-line allele information was available, but the variant has been reported ‘Confirmed Somatic’ in a normal–tumor sample pair from another patient. Moreover, concerning single point mutations, we included in our analysis only mutations present in tumor samples that are previously reported or confirmed to be somatic and are predicted to be pathogenic by the FATHMM algorithm.

**Figure 5:**
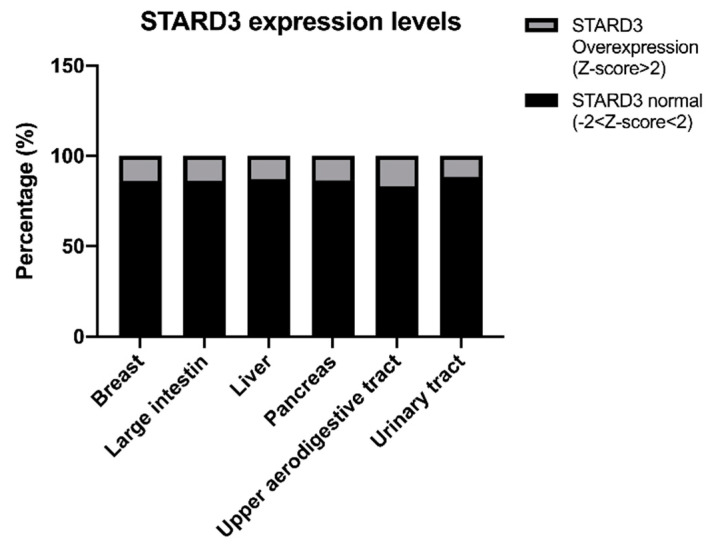
STARD3 expression levels in cancers.

Overall, the analysis revealed the existence of 88 somatic mutations affecting STARD3. The results showed that mutations are distributed over the entire length of the protein, with a slight prevalence in the START domain (49%) compared with the MENTAL domain (38.6%) (Supplementary Table S1). The most frequent mutation is present in residue 117 (R117Q) in 5 patients with adenocarcinoma (3 in the intestine and 2 in the stomach). The same mutation is also reported in thyroid and prostate cancer, but tumor samples are not specified; for this reason, they were not included in the analysis. Only one pathogenic mutation (0.99 predicted score) is present in the FFAT-like motif of the protein. This missense mutation leads to the replacement of the amino acid serine with leucine at position 209 and is present in a dedifferentiated liposarcoma. It has been reported by Di Mattia et al. that the phosphorylation of this serine (4th residue of the FFAT motif) of STARD3 is necessary for ER–endosome contact formation in vivo and for sterol transfer function in vitro [169]. It is, therefore, possible to hypothesize that when this amino acid is mutated to leucine there is an abrogation of ER–endosome contact sites since it was previously reported that the substitution of the other amino acids of the FFAT-like motif (residue 207 and 208) abrogates ER–endosome contacts [42]. Regarding gene expression levels, our analysis reported 404 tumor samples where STARD3 is overexpressed with a Z-score level higher than 2. Of relevance are the percentages of some tumor types in which STARD3 is overexpressed: breast cancer (123 out of 883 samples; 13.9%), large intestine cancer (25 out of 182 samples; 13.7%), liver cancer (29 out of 224 samples; 12.9%), pancreatic cancer (15 out of 109 samples; 13.76%), upper aerodigestive tract cancer (20 out of 118 samples; 16.9%), urinary tract cancer (35 out of 303 samples; 11.5%) (Figure 5).

## 7. STARD3 Inhibitors

Until now, there has only been one STARD3 inhibitor reported in the literature. The binding interactions of cholesterol inside STARD3 provided by Lapillo et al. [43] led to the definition of a structure-based pharmacophore model generated with LigandScout [178], which was used for virtual screening (VS) studies. Approximately 1,700,000 molecules were filtered using this approach and only the 5456 molecules that showed the double H-bond donor/acceptor feature and at least three other features were subjected to a CD protocol.

**Figure 6:**
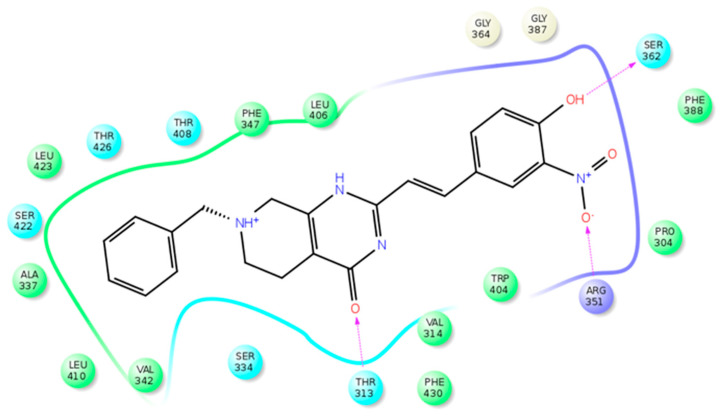
Schematic view of the STARD3–VS1 interactions. Polar, charged (positive), and hydrophobic residues are colored light blue, violet, and green, respectively. H-bonds are showed as arrows, with the arrowhead that indicates the H-bond acceptor portion.

After a virtual screening approach, 42 compounds showed a good level of consensus and were subjected to MD simulations to assess the stability of their predicted binding. After the analysis, eight compounds were tested for their ability to inhibit the STARD3 activity. Only compound VS1 showed good activity (IC50 of 35 µM). Figure 6 shows a schematic 2D representation of the main interactions of this compound inside STARD3. The amino acids T313, V314, A337, R351, S362, W404, L406, T408, L410, L423, T426, and F430 are involved in the interaction with VS1 and details were reported in [43].

On the other hand, Chitrala et al. used a ligand-based virtual screening to identify ligands against ZINC (727 842 molecules with 3D structures). The D (-)-TAR inhibitor (PDB code: 1EM2) in complex with the crystal structure 3D coordinates of the START domain of STARD3, was selected from the Protein Databank (PDB) as the receptor model. Only three molecules were found to have high affinity in vitro as potent inhibitors of STARD3, indicating an alternative use of this technique in the drug discovery process [179].

## 8. Prospective and Conclusions

Cancer cells are characterized by uncontrolled cell growth, migration, and invasion, as well as high lipid metabolism. Many of these processes are influenced by lipid-transfer proteins (LTPs), which can modulate the lipid levels, including cholesterol, and thus, modify diverse signaling pathways.

START proteins are a family of LTP involved in non-vesicular cholesterol transport. These proteins play an important function in intracellular cholesterol distribution and are associated with the development of different cancer types. Thus, the identification of specific START proteins that induce development of neoplastic pathologies could be utilized for therapeutic interventions.

The data discussed herein suggest that STARD3 has a role on cholesterol transportation in tumor cells. In different types of cancers, such as steroid hormone-driven cancers (breast and prostate cancers), the amplification/overexpression of STARD3 could promote intra-neoplastic autonomous steroidogenesis and contribute primarily to the development of the malignancy. In-depth molecular studies are needed to clarify which protein partners cooperate with STARD3 in each specific cancer type and to identify the molecular networks that could act to compensate STARD3 alterations for better therapeutic approaches.

The development of anti-STARD3-targeted therapies represents a promising strategy for cancer patients with elevated STARD3 expression. Up to now, only one inhibitor has been developed by our group. The VS1 compound is able to inhibit cholesterol interactions with STARD3 in the low micromolar range. This compound represents a starting point for the development of novel STARD3-targeted therapeutic agents with high selectivity and potency in the nanomolar range.

## Figures and Tables

**Table 1:** Summary of START proteins and their alteration in human diseases.

Name	Alias	Function	Disease	Description	References
STARD1	StAR	Cholesterol transport	Congenital lipoid adrenal hyperplasia	Mutations induced a failure to transport cholesterol to the inner mitochondria membrane	[93]
STARD2	PCTP-like	Lipid (phosphatidylcholine) transport	Diabetes	Functional inactivation decreases hepatic glucose production	[94]
STARD3	MLN64	Cholesterol transport	BC	Overexpression and amplification in breast cancer (BC)	[45,95]
STARD4	START only	Cholesterol transport	BC	Overexpression is associated with shorter, distant, metastasis-free survival time	[96]
STARD5	START only	Cholesterol transport	Colon cancer; Diabetes	Overexpression in colon cancer; overexpressed in kidneys of a diabetic mouse model	[97,98]
STARD7	GTT1	Lipid (phosphatidylcholine) transport	Cancer	Overexpression in trophoblast cancer, colorectal cancer, B chronic lymphatic leukemia	[99,100,101,102,103]
STARD8	DLC3	Stimulates the hydrolysis of phosphatidylinositol	Cancer/metastasis	Overexpression inhibits tumor growth and progression	[104,105,106,107]
STARD10	PCTP-like	Lipid (phosphatidylcholine and phosphatidylethanolamine) transport	BC; Diabetes	Overexpression in BC; regulation of insulin secretion	[108,109,110,111,112]
STARD11	CERT	Ceramide transport	Cancer	Overexpression in HER2+ breast cancer and downexpression in triple-negative BC	[113,114]
STARD12	DLC1	GTPase-activating protein	Cancer	Deletion in liver cancer; downregulation in lung, BC, colon, and prostate cancer	[106,115,116,117]
STARD13	DLC2	GTPase-activating protein	Cancer	Downregulation in lung, breast, ovarian, uterine, renal, gastric, rectal, colon, glioma, and liver tumors	[106,118,119,120,121]
STARD15	ACOT12	acyl-CoA thioesterase 12	Hepatocellular cancer (HCC)	Downregulation and associated with HCC metastasis and poor survival of HCC patients	[122]

## Supplementary Materials

The following are available online at https://www.mdpi.com/article/10.3390/cancers13184693/s1, Table S1: Predicted pathogenic mutations in STARD3.

## Abbreviations

START	Steroidogenic Acute Regulatory transfer
STARD3	STAR-related lipid transfer domain-3
FDA	Food and Drug Administration
CDKs	cyclin-dependent kinase
EGFR	epidermal growth factor receptor
VEGF	vascular endothelial growth factor
PARP	poly (ADP-ribose) polymerase
PD-1	programmed death-1
PD-L1	programmed death-ligand 1
LTP	lipid transfer protein
LDL	low-density lipoprotein
MLN64	metastatic lymph node clone 64 protein
MENTAL	(MLN64 NH (2)-terminal domain)
ER	Endoplasmic Reticulum
LE	Late Endosome
VAP	vesicle-associated membrane-protein-associated protein
MSP	Major sperm protein domain
MCS	Membrane contact sites
MD	molecular dynamic
CD	Consensus docking

## References

[1] Siegel R.L., Miller K.D., Jemal A. (2020). Cancer statistics, 2020. CA Cancer J. Clin..

[2] Danaei G., Vander Hoorn S., Lopez A.D., Murray C.J.L., Ezzati M. (2005). Causes of cancer in the world: Comparative risk assessment of nine behavioural and environmental risk factors. Lancet.

[3] Bray F., Ferlay J., Soerjomataram I., Siegel R.L., Torre L.A., Jemal A. (2018). Global cancer statistics 2018: GLOBOCAN estimates of incidence and mortality worldwide for 36 cancers in 185 countries. CA Cancer J. Clin..

[4] Ferlay J., Soerjomataram I., Dikshit R., Eser S., Mathers C., Rebelo M., Parkin D.M., Forman D., Bray F. (2015). Cancer incidence and mortality worldwide: Sources, methods and major patterns in GLOBOCAN 2012. Int. J. Cancer.

[5] Katzke V.A., Kaaks R., Kühn T. (2015). Lifestyle and cancer risk. Cancer J..

[6] Ferlay J., Colombet M., Soerjomataram I., Dyba T., Randi G., Bettio M., Gavin A., Visser O., Bray F. (2018). Cancer incidence and mortality patterns in Europe: Estimates for 40 countries and 25 major cancers in 2018. Eur. J. Cancer.

[7] de Castro Sant’ Anna C., Junior A.G.F., Soares P., Tuji F., Paschoal E., Chaves L.C., Burbano R.R. (2018). Molecular biology as a tool for the treatment of cancer. Clin. Exp. Med..

[8] Liu Z., Lin Y., Zhang J., Zhang Y., Li Y., Liu Z., Li Q., Luo M., Liang R., Ye J. (2019). Molecular targeted and immune checkpoint therapy for advanced hepatocellular carcinoma. J. Exp. Clin. Cancer Res..

[9] Pellino A., Riello E., Nappo F., Brignola S., Murgioni S., Djaballah S.A., Lonardi S., Zagonel V., Rugge M., Loupakis F. (2019). Targeted therapies in metastatic gastric cancer: Current knowledge and future perspectives. World J. Gastroenterol..

[10] Patil T., Simons E., Mushtaq R., Pacheco J.M., Doebele R.C., Bowles D.W. (2019). Targeted therapies for ROS1-rearranged non-small cell lung cancer. Drugs Today.

[11] Dabney R.S., Khalife M., Shahid K., Phan A.T. (2019). Molecular pathways and targeted therapy in cholangiocarcinoma. Clin. Adv. Hematol. Oncol..

[12] Lee Y.T., Tan Y.J., Oon C.E. (2018). Molecular targeted therapy: Treating cancer with specificity. Eur. J. Pharmacol..

[13] Saijo N. (2010). Progress in cancer chemotherapy with special stress on molecular-targeted therapy. Jpn. J. Clin. Oncol..

[14] Hideshima T., Qi J., Paranal R.M., Tang W., Greenberg E., West N., Colling M.E., Estiu G., Mazitschek R., Perry J.A. (2016). Discovery of selective small-molecule HDAC6 inhibitor for overcoming proteasome inhibitor resistance in multiple myeloma. Proc. Natl. Acad. Sci. USA.

[15] Tripathy D., Bardia A., Sellers W.R. (2017). Ribociclib (LEE011): Mechanism of Action and Clinical Impact of This Selective Cyclin-Dependent Kinase 4/6 Inhibitor in Various Solid Tumors. Clin. Cancer Res..

[16] Hoelder S., Clarke P.A., Workman P. (2012). Discovery of small molecule cancer drugs: Successes, challenges and opportunities. Mol. Oncol..

[17] Tirumani S.H., Fairchild A., Krajewski K.M., Nishino M., Howard S.A., Baheti A.D., Rosenthal M.H., Jagannathan J.P., Shinagare A.B., Ramaiya N.H. (2015). Weanti-VEGF molecular targeted therapies in common solid malignancies: Comprehensive update for radiologists. Radiographics.

[18] Balasubramaniam S., Beaver J.A., Horton S., Fernandes L.L., Tang S., Horne H.N., Liu J., Liu C., Schrieber S.J., Yu J. (2017). FDA approval summary: Rucaparib for the treatment of patients with deleterious BRCA mutation–associated advanced ovarian cancer. Clin. Cancer Res..

[19] Essel K.G., Moore K.N. (2018). Niraparib for the treatment of ovarian cancer. Expert Rev. Anticancer Ther..

[20] Hoy S.M. (2019). Sintilimab: First Global Approval. Drugs.

[21] Kuzu O.F., Noory M.A., Robertson G.P. (2016). The role of cholesterol in cancer. Cancer Res..

[22] Ding X., Zhang W., Li S., Yang H. (2019). The role of cholesterol metabolism in cancer. Am. J. Cancer Res..

[23] Mollinedo F., Gajate C. (2015). Lipid rafts as major platforms for signaling regulation in cancer. Adv. Biol. Regul..

[24] Murai T. (2015). Cholesterol lowering: Role in cancer prevention and treatment. Biol. Chem..

[25] Mollinedo F., Gajate C. (2020). Lipid rafts as signaling hubs in cancer cell survival/death and invasion: Implications in tumor progression and therapy. J. Lipid Res..

[26] Zhuang L., Kim J., Adam R.M., Solomon K.R., Freeman M.R. (2005). Cholesterol targeting alters lipid raft composition and cell survival in prostate cancer cells and xenografts. J. Clin. Investig..

[27] Amemiya-Kudo M., Shimano H., Hasty A.H., Yahagi N., Yoshikawa T., Matsuzaka T., Okazaki H., Tamura Y., Iizuka Y., Ohashi K. (2002). Transcriptional activities of nuclear SREBP-1a, -1c, and -2 to different target promoters of lipogenic and cholesterogenic genes. J. Lipid Res..

[28] Yang T., Espenshade P.J., Wright M.E., Yabe D., Gong Y., Aebersold R., Goldstein J.L., Brown M.S. (2002). Crucial step in cholesterol homeostasis: Sterols promote binding of SCAP to INSIG-1, a membrane protein that facilitates retention of SREBPs in ER. Cell.

[29] Wüstner D., Solanko K. (2015). How cholesterol interacts with proteins and lipids during its intracellular transport. Biochim. Biophys. Acta—Biomembr..

[30] Yabe D., Brown M.S., Goldstein J.L. (2002). Insig-2, a second endoplasmic reticulum protein that binds SCAP and blocks export of sterol regulatory element-binding proteins. Proc. Natl. Acad. Sci. USA.

[31] Sun L.P., Li L., Goldstein J.L., Brown M.S. (2005). Insig required for sterol-mediated inhibition of Scap/SREBP binding to COPII proteins in vitro. J. Biol. Chem..

[32] Nemecz G., Schroeder F. (1991). Selective binding of cholesterol by recombinant fatty acid binding proteins. J. Biol. Chem..

[33] Raychaudhuri S., Prinz W.A. (2010). The diverse functions of oxysterol-binding proteins. Annu. Rev. Cell Dev. Biol..

[34] Naito T., Ercan B., Krshnan L., Triebl A., Koh D.H.Z., Wei F.Y., Tomizawa K., Torta F.T., Wenk M.R., Saheki Y. (2019). Movement of accessible plasma membrane cholesterol by GRAMD1 lipid transfer protein complex. Elife.

[35] Enrich C., Rentero C., Grewal T., Futter C.E., Eden E.R. (2019). Cholesterol Overload: Contact Sites to the Rescue!. Contact.

[36] Storch J., Xu Z. (2009). Niemann-Pick C2 (NPC2) and intracellular cholesterol trafficking. Biochim. Biophys. Acta—Mol. Cell Biol. Lipids.

[37] Mesmin B., Antonny B., Drin G. (2013). Insights into the mechanisms of sterol transport between organelles. Cell. Mol. Life Sci..

[38] Iaea D.B., Mao S., Maxfield F.R. (2014). Steroidogenic acute regulatory protein-related lipid transfer (START) proteins in non-vesicular cholesterol transport. Cholesterol Transporters of the START Domain Protein Family in Health and Disease.

[39] Soccio R.E., Adams R.M., Romanowski M.J., Sehayek E., Burley S.K., Breslow J.L. (2002). The cholesterol-regulated StarD4 gene encodes a StAR-related lipid transfer protein with two closely related homologues, StarD5 and StarD6. Proc. Natl. Acad. Sci. USA.

[40] Liapis A., Chen F.W., Davies J.P., Wang R., Ioannou Y.A. (2012). MLN64 transport to the late endosome is regulated by binding to 14-3-3 via a non-canonical binding site. PLoS ONE.

[41] Hölttä-Vuori M., Alpy F., Tanhuanpää K., Jokitalo E., Mutka A.L., Ikonen E. (2005). MLN64 is involved in actin-mediated dynamics of late endocytic organelles. Mol. Biol. Cell.

[42] Wilhelm L.P., Wendling C., Védie B., Kobayashi T., Chenard M., Tomasetto C., Drin G., Alpy F. (2017). STARD 3 mediates endoplasmic reticulum-to-endosome cholesterol transport at membrane contact sites. EMBO J..

[43] Lapillo M., Salis B., Palazzolo S., Poli G., Granchi C., Minutolo F., Rotondo R., Caligiuri I., Canzonieri V., Tuccinardi T. (2019). First-of-its-kind STARD3 Inhibitor: In Silico Identification and Biological Evaluation as Anticancer Agent. ACS Med. Chem. Lett..

[44] Vassilev B., Sihto H., Li S., Hölttä-Vuori M., Ilola J., Lundin J., Isola J., Kellokumpu-Lehtinen P.L., Joensuu H., Ikonen E. (2015). Elevated levels of StAR-related lipid transfer protein 3 alter cholesterol balance and adhesiveness of breast cancer cells: Potential mechanisms contributing to progression of HER2-positive breast cancers. Am. J. Pathol..

[45] Alpy F., Boulay A., Moog-Lutz C., Andarawewa K.L., Degot S., Stoll I., Rio M.C., Tomasetto C. (2003). Metastatic lymph node 64 (MLN64), a gene overexpressed in breast cancers, is regulated by Sp/KLF transcription factors. Oncogene.

[46] Stigliano A., Gandini O., Cerquetti L., Gazzaniga P., Misiti S., Monti S., Gradilone A., Falasca P., Poggi M., Brunetti E. (2007). Increased metastatic lymph node 64 and CYP17 expression are associated with high stage prostate cancer. J. Endocrinol..

[47] Cai W., Ye L., Sun J., Mansel R.E., Jiang W.G. (2010). Expression of MLN64 influences cellular matrix adhesion of breast cancer cells, the role for focal adhesion kinase. Int. J. Mol. Med..

[48] Vinatzer U., Dampier B., Streubel B., Pacher M., Seewald M.J., Stratowa C., Kaserer K., Schreiber M. (2005). Expression of HER2 and the coamplified genes GRB7 and MLN64 in human breast cancer: Quantitative real-time reverse transcription-PCR as a diagnostic alternative to immunohistochemistry and fluorescence in situ hybridization. Clin. Cancer Res..

[49] Katoh M., Katoh M. (2004). Evolutionary recombination hotspot around GSDML-GSDM locus is closely linked to the oncogenomic recombination hotspot around the PPP1R1B-ERBB2-GRB7 amplicon. Int. J. Oncol..

[50] Qiu Y., Zhang Z.Y., Du W.D., Ye L., Xu S., Zuo X.B., Zhou F.S., Chen G., Ma X.L., Schneider M.E. (2014). Association analysis of ERBB2 amplicon genetic polymorphisms and STARD3 expression with risk of gastric cancer in the chinese population. Gene.

[51] Muzny D.M., Bainbridge M.N., Chang K., Dinh H.H., Drummond J.A., Fowler G., Kovar C.L., Lewis L.R., Morgan M.B., Newsham I.F. (2012). Comprehensive molecular characterization of human colon and rectal cancer. Nature.

[52] Tuccinardi T., Poli G., Corchia I., Granchi C., Lapillo M., Macchia M., Minutolo F., Ortore G., Martinelli A. (2016). A Virtual Screening Study for Lactate Dehydrogenase 5 Inhibitors by Using a Pharmacophore-based Approach. Mol. Inform..

[53] Shafique K., McLoone P., Qureshi K., Leung H., Hart C., Morrison D.S. (2012). Cholesterol and the risk of grade-specific prostate cancer incidence: Evidence from two large prospective cohort studies with up to 37 years’ follow up. BMC Cancer.

[54] Allott E.H., Howard L.E., Cooperberg M.R., Kane C.J., Aronson W.J., Terris M.K., Amling C.L., Freedland S.J. (2014). Serum lipid profile and risk of prostate cancer recurrence: Results from the SEARCH database. Cancer Epidemiol. Biomark. Prev..

[55] Mamtani R., Lewis J.D., Scott F.I., Ahmad T., Goldberg D.S., Datta J., Yang Y.X., Boursi B. (2016). Disentangling the Association between Statins, Cholesterol, and Colorectal Cancer: A Nested Case-Control Study. PLoS Med..

[56] Wang C., Li P., Xuan J., Zhu C., Liu J., Shan L., Du Q., Ren Y., Ye J. (2017). Cholesterol Enhances Colorectal Cancer Progression via ROS Elevation and MAPK Signaling Pathway Activation. Cell. Physiol. Biochem..

[57] Lin X., Liu L., Fu Y., Gao J., He Y., Wu Y., Lian X. (2018). Dietary cholesterol intake and risk of lung cancer: A meta-analysis. Nutrients.

[58] Lyu Z., Li N., Wang G., Su K., Li F., Guo L., Feng X., Wei L., Chen H., Chen Y. (2018). Association between total cholesterol and risk of lung cancer incidence in men: A prospective cohort study. Chin. J. Endem..

[59] Nelson E.R. (2018). The significance of cholesterol and its metabolite, 27-hydroxycholesterol in breast cancer. Mol. Cell. Endocrinol..

[60] Touvier M., Fassier P., His M., Norat T., Chan D.S.M., Blacher J., Hercberg S., Galan P., Druesne-Pecollo N., Latino-Martel P. (2015). Cholesterol and breast cancer risk: A systematic review and meta-analysis of prospective studies. Br. J. Nutr..

[61] Jacobs E.J., Newton C.C., Thun M.J., Gapstur S.M. (2011). Long-term use of cholesterol-lowering drugs and cancer incidence in a large United States cohort. Cancer Res..

[62] Cardwell C.R., Hicks B.M., Hughes C., Murray L.J. (2014). Statin Use after colorectal cancer diagnosis and survival: A population-based cohort study. J. Clin. Oncol..

[63] Murtola T.J., Visvanathan K., Artama M., Vainio H., Pukkala E. (2014). Statin use and breast cancer survival: A nationwide cohort study from Finland. PLoS ONE.

[64] Nielsen S.F., Nordestgaard B.G., Bojesen S.E. (2012). Statin Use and Reduced Cancer-Related Mortality. N. Engl. J. Med..

[65] Cattley R.C. (1996). Carcinogenicity of lipid-lowering drugs. JAMA J. Am. Med. Assoc..

[66] Freed-Pastor W.A., Mizuno H., Zhao X., Langerød A., Moon S.H., Rodriguez-Barrueco R., Barsotti A., Chicas A., Li W., Polotskaia A. (2012). Mutant p53 disrupts mammary tissue architecture via the mevalonate pathway. Cell.

[67] Swinnen J.V., Brusselmans K., Verhoeven G. (2006). Increased lipogenesis in cancer cells: New players, novel targets. Curr. Opin. Clin. Nutr. Metab. Care.

[68] Krause M.R., Regen S.L. (2014). The structural role of cholesterol in cell membranes: From condensed bilayers to lipid rafts. Acc. Chem. Res..

[69] Maxfield F.R., van Meer G. (2010). Cholesterol, the central lipid of mammalian cells. Curr. Opin. Cell Biol..

[70] Goldstein J.L., Brown M.S. (2009). The LDL receptor. Arterioscler. Thromb. Vasc. Biol..

[71] Iaea D.B., Maxfield F.R. (2015). Cholesterol trafficking and distribution. Essays Biochem..

[72] Hao M., Lin S.X., Karylowski O.J., Stner D.W., McGraw T.E., Maxfield F.R. (2002). Vesicular and non-vesicular sterol transport in living cells: The endocytic recycling compartment is a major sterol storage organelle. J. Biol. Chem..

[73] D’Angelo G., Vicinanza M., De Matteis M.A. (2008). Lipid-transfer proteins in biosynthetic pathways. Curr. Opin. Cell Biol..

[74] Das K., Nozaki T. (2018). Non-vesicular lipid transport machinery in Entamoeba histolytica. Front. Cell. Infect. Microbiol..

[75] Prinz W.A. (2007). Non-vesicular sterol transport in cells. Prog. Lipid Res..

[76] Chimento A., Casaburi I., Avena P., Trotta F., De Luca A., Rago V., Pezzi V., Sirianni R. (2019). Cholesterol and its metabolites in tumor growth: Therapeutic potential of statins in cancer treatment. Front. Endocrinol..

[77] Li Y.C., Park M.J., Ye S.K., Kim C.W., Kim Y.N. (2006). Elevated levels of cholesterol-rich lipid rafts in cancer cells are correlated with apoptosis sensitivity induced by cholesterol-depleting agents. Am. J. Pathol..

[78] Levin-Gromiko U., Koshelev V., Kushnir P., Fedida-Metula S., Voronov E., Fishman D. (2014). Amplified lipid rafts of malignant cells constitute a target for inhibition of aberrantly active NFAT and melanoma tumor growth by the aminobisphosphonate zoledronic acid. Carcinogenesis.

[79] De Berardinis R.J., Chandel N.S. (2016). Fundamentals of cancer metabolism. Sci. Adv..

[80] Currie E., Schulze A., Zechner R., Walther T.C., Farese R.V. (2013). Cellular fatty acid metabolism and cancer. Cell Metab..

[81] Ward P.S., Thompson C.B. (2012). Metabolic Reprogramming: A Cancer Hallmark Even Warburg Did Not Anticipate. Cancer Cell.

[82] Cairns R.A., Harris I., Mccracken S., Mak T.W. (2011). Cancer cell metabolism. Cold Spring Harb. Symp. Quant. Biol..

[83] Badana A.K., Chintala M., Gavara M.M., Naik S., Kumari S., Kappala V.R., Iska B.R., Malla R.R. (2018). Lipid rafts disruption induces apoptosis by attenuating expression of LRP6 and survivin in triple negative breast cancer. Biomed. Pharmacother..

[84] Raghu H., Sodadasu P.K., Malla R.R., Gondi C.S., Estes N., Rao J.S. (2010). Localization of uPAR and MMP-9 in lipid rafts is critical for migration, invasion and angiogenesis in human breast cancer cells. BMC Cancer.

[85] Staubach S., Hanisch F.G. (2011). Lipid rafts: Signaling and sorting platforms of cells and their roles in cancer. Expert Rev. Proteomics.

[86] Porstmann T., Santos C.R., Griffiths B., Cully M., Wu M., Leevers S., Griffiths J.R., Chung Y.L., Schulze A. (2008). SREBP Activity Is Regulated by mTORC1 and Contributes to Akt-Dependent Cell Growth. Cell Metab..

[87] Thysell E., Surowiec I., Hörnberg E., Crnalic S., Widmark A., Johansson A.I., Stattin P., Bergh A., Moritz T., Antti H. (2010). Metabolomic characterization of human prostate cancer bone metastases reveals increased levels of cholesterol. PLoS ONE.

[88] Yue S., Li J., Lee S.Y., Lee H.J., Shao T., Song B., Cheng L., Masterson T.A., Liu X., Ratliff T.L. (2014). Cholesteryl ester accumulation induced by PTEN loss and PI3K/AKT activation underlies human prostate cancer aggressiveness. Cell Metab..

[89] Clark B.J. (2012). The mammalian START domain protein family in lipid transport in health and disease. J. Endocrinol..

[90] Alpy F., Tomasetto C. (2014). START ships lipids across interorganelle space. Biochimie.

[91] Alpy F., Tomasetto C. (2005). Give lipids a START: The StAR-related lipid transfer (START) domain in mammals. J. Cell Sci..

[92] Soccio R.E., Breslow J.L. (2003). StAR-related lipid transfer (START) proteins: Mediators of intracellular lipid metabolism. J. Biol. Chem..

[93] Stocco D.M. (2002). Clinical disorders associated with abnormal cholesterol transport: Mutations in the steroidogenic acute regulatory protein. Mol. Cell. Endocrinol..

[94] Shishova E.Y., Stoll J.M., Ersoy B.A., Shrestha S., Scapa E.F., Li Y., Niepel M.W., Su Y., Jelicks L.A., Stahl G.L. (2011). Genetic ablation or chemical inhibition of phosphatidylcholine transfer protein attenuates diet-induced hepatic glucose production. Hepatology.

[95] Moog-Lutz C., Tomasetto C., Régnier C.H., Wendling C., Lutz Y., Muller D., Chenard M.P., Basset P., Rio M.C. (1997). MLN64 exhibits homology with the steroidogenic acute regulatory protein (STAR) and is over-expressed in human breast carcinomas. Int. J. Cancer.

[96] Zhang M., Xiang Z., Wang F., Shan R., Li L., Chen J., Liu B.A., Huang J., Sun L.Q., Zhou W.B. (2020). STARD4 promotes breast cancer cell malignancy. Oncol. Rep..

[97] Chen Y.C., Meier R.K., Zheng S., Khundmiri S.J., Tseng M.T., Lederer E.D., Epstein P.N., Clark B.J. (2009). Steroidogenic acute regulatory-related lipid transfer domain protein 5 localization and regulation in renal tubules. Am. J. Physiol.-Ren. Physiol..

[98] Sharon C., Boothello R.S., Rodriguez-Agudo D., Gill G., Pandak W.M., Patel B.B. (2018). Sa1196—Steroidogenic Acute Regulatory Protein (Start) Related Lipid Transfer Domain Containing 5 (Stard5) is a Novel Target for Colon Cancer Stem Cells. Gastroenterology.

[99] Angeletti S., Rena V., Nores R., Fretes R., Panzetta-Dutari G.M., Genti-Raimondi S. (2008). Expression and Localization of StarD7 in Trophoblast Cells. Placenta.

[100] Durand S., Angeletti S., Genti-Raimondi S. (2004). GTT1/StarD7, a novel phosphatidylcholine transfer protein-like highly expressed in gestational trophoblastic tumour: Cloning and characterization. Placenta.

[101] Flores-Martín J., Rena V., Márquez S., Panzetta-Dutari G.M., Genti-Raimondi S. (2012). StarD7 Knockdown Modulates ABCG2 Expression, Cell Migration, Proliferation, and Differentiation of Human Choriocarcinoma JEG-3 Cells. PLoS ONE.

[102] Wiese A.H., Auer J., Lassmann S., Nährig J., Rosenberg R., Höfler H., Rüger R., Werner M. (2007). Identification of gene signatures for invasive colorectal tumor cells. Cancer Detect. Prev..

[103] Jelinek D.F., Tschumper R.C., Stolovitzky G.A., Iturria S.J., Tu Y., Lepre J., Shah N., Kay N.E. (2003). Identification of a global gene expression signature of B-chronic lymphocytic leukemia. Mol. Cancer Res..

[104] Kawai K., Kiyota M., Seike J., Deki Y., Yagisawa H. (2007). START-GAP3/DLC3 is a GAP for RhoA and Cdc42 and is localized in focal adhesions regulating cell morphology. Biochem. Biophys. Res. Commun..

[105] Kawai K., Iwamae Y., Yamaga M., Kiyota M., Ishii H., Hirata H., Homma Y., Yagisawa H. (2009). Focal adhesion-localization of START-GAP1/DLC1 is essential for cell motility and morphology. Genes Cells.

[106] Sun L., Sun J., Song J.D. (2019). High expression of DLC family proteins predicts better prognosis and inhibits tumor progression in NSCLC. Mol. Med. Rep..

[107] Durkin M.E., Ullmannova V., Guan M., Popescu N.C. (2007). Deleted in liver cancer 3 (DLC-3), a novel Rho GTPase-activating protein, is downregulated in cancer and inhibits tumor cell growth. Oncogene.

[108] Olayioye M.A., Vehring S., Müller P., Herrmann A., Schiller J., Thiele C., Lindeman G.J., Visvader J.E., Pomorski T. (2005). StarD10, a START domain protein overexpressed in breast cancer, functions as a phospholipid transfer protein. J. Biol. Chem..

[109] Olayioye M.A., Buchholz M., Schmid S., Schöffler P., Hoffmann P., Pomorski T. (2007). Phosphorylation of StarD10 on serine 284 by casein kinase II modulates its lipid transfer activity. J. Biol. Chem..

[110] Floris A., Luo J., Frank J., Zhou J., Orrù S., Biancolella M., Pucci S., Orlandi A., Campagna P., Balzano A. (2019). Star-related lipid transfer protein 10 (STARD10): A novel key player in alcohol-induced breast cancer progression 11 Medical and Health Sciences 1112 Oncology and Carcinogenesis. J. Exp. Clin. Cancer Res..

[111] Carrat G.R., Hu M., Nguyen-Tu M.S., Chabosseau P., Gaulton K.J., van de Bunt M., Siddiq A., Falchi M., Thurner M., Canouil M. (2017). Decreased STARD10 Expression Is Associated with Defective Insulin Secretion in Humans and Mice. Am. J. Hum. Genet..

[112] Carrat G.R., Haythorne E., Tomas A., Haataja L., Müller A., Arvan P., Piunti A., Cheng K., Huang M., Pullen T.J. (2020). The type 2 diabetes gene product STARD10 is a phosphoinositide-binding protein that controls insulin secretory granule biogenesis. Mol. Metab..

[113] Heering J., Weis N., Holeiter M., Neugart F., Staebler A., Fehm T.N., Bischoff A., Schiller J., Duss S., Schmid S. (2012). Loss of the ceramide transfer protein augments EGF receptor signaling in breast cancer. Cancer Res..

[114] Lee A.J.X., Roylance R., Sander J., Gorman P., Endesfelder D., Kschischo M., Jones N.P., East P., Nicke B., Spassieva S. (2012). CERT depletion predicts chemotherapy benefit and mediates cytotoxic and polyploid-specific cancer cell death through autophagy induction. J. Pathol..

[115] Yuan B.Z., Zhou X., Durkin M.E., Zimonjic D.B., Gumundsdottir K., Eyfjord J.E., Thorgeirsson S.S., Popescu N.C. (2003). DLC-1 gene inhibits human breast cancer cell growth and in vivo tumorigenicity. Oncogene.

[116] Wong C.M., Lee J.M.F., Ching Y.P., Jin D.Y., Ng I.O.L. (2003). Genetic and Epigenetic Alterations of DLC-1 Gene in Hepatocellular Carcinoma. Cancer Res..

[117] Ng I.O.L., Liang Z.D., Cao L., Lee T.T.K.W. (2000). DLC-1 is deleted in primary hepatocellular carcinoma and exerts inhibitory effects on the proliferation of hepatoma cell lines with deleted DLC-1. Cancer Res..

[118] Basak P., Leslie H., Dillon R.L., Muller W.J., Raouf A., Mowat M.R.A. (2018). In vivo evidence supporting a metastasis suppressor role for Stard13 (Dlc2) in ErbB2 (Neu) oncogene induced mouse mammary tumors. Genes Chromosom. Cancer.

[119] Yang Z., Chen H., Shu M.A.N., Zhang Y., Xue L., Lin Y. (2019). DLC2 operates as a tumor suppressor gene in breast cancer via the RhoGTPase pathway. Oncol. Lett..

[120] Cheng C., Feng S., Jiao J., Huang W., Huang J., Wang L., Jiang W., Jiang C., Dai M., Li Z. (2018). DLC2 inhibits development of glioma through regulating the expression ratio of TAp73alpha/TAp73beta. Am. J. Cancer Res..

[121] Wolosz D., Walczak A., Szparecki G., Dwojak M., Winiarska M., Wolinska E., Gornicka B. (2019). Deleted in liver cancer 2 (DLC2) protein expression in hepatocellular carcinoma. Eur. J. Histochem..

[122] Lu M., Zhu W.W., Wang X., Tang J.J., Zhang K.L., Yu G.Y., Shao W.Q., Lin Z.F., Wang S.H., Lu L. (2019). ACOT12-Dependent Alteration of Acetyl-CoA Drives Hepatocellular Carcinoma Metastasis by Epigenetic Induction of Epithelial-Mesenchymal Transition. Cell Metab..

[123] Rodriguez-Agudo D., Ren S., Hylemon P.B., Redford K., Natarajan R., Del Castillo A., Gil G., Pandak W.M. (2005). Human StarD5, a cytosolic StAR-related lipid binding protein. J. Lipid Res..

[124] Rodriguez-Agudo D., Malacrida L., Kakiyama G., Sparrer T., Fortes C., Maceyka M., Subler M.A., Windle J.J., Gratton E., Pandak W.M. (2019). StarD5: An ER stress protein regulates plasma membrane and intracellular cholesterol homeostasis. J. Lipid Res..

[125] Yang L., Na C.L., Luo S., Wu D., Hogan S., Huang T., Weaver T.E. (2017). The Phosphatidylcholine Transfer Protein Stard7 is Required for Mitochondrial and Epithelial Cell Homeostasis. Sci. Rep..

[126] Flores-Martin J., Rena V., Angeletti S., Panzetta-Dutari G.M., Genti-Raimondi S. (2013). The lipid transfer protein StarD7: Structure, function, and regulation. Int. J. Mol. Sci..

[127] Torres J.Z. (2012). STARD9/Kif16a is a novel mitotic kinesin and antimitotic target. Bioarchitecture.

[128] Srivastava S., Panda D. (2018). A centrosomal protein STARD9 promotes microtubule stability and regulates spindle microtubule dynamics. Cell Cycle.

[129] Olayioye M.A., Hoffmann P., Pomorski T., Armes J., Simpson R.J., Kemp B.E., Lindeman G.J., Visvader J.E. (2004). The phosphoprotein StarD10 is overexpressed in breast cancer and cooperates with ErbB receptors in cellular transformation. Cancer Res..

[130] Hanada K., Kumagai K., Tomishige N., Yamaji T. (2009). CERT-mediated trafficking of ceramide. Biochim. Biophys. Acta—Mol. Cell Biol. Lipids.

[131] Juul N., Szallasi Z., Eklund A.C., Li Q., Burrell R.A., Gerlinger M., Valero V., Andreopoulou E., Esteva F.J., Symmans W.F. (2010). Assessment of an RNA interference screen-derived mitotic and ceramide pathway metagene as a predictor of response to neoadjuvant paclitaxel for primary triple-negative breast cancer: A retrospective analysis of five clinical trials. Lancet Oncol..

[132] Swanton C., Marani M., Pardo O., Warne P.H., Kelly G., Sahai E., Elustondo F., Chang J., Temple J., Ahmed A.A. (2007). Regulators of Mitotic Arrest and Ceramide Metabolism Are Determinants of Sensitivity to Paclitaxel and Other Chemotherapeutic Drugs. Cancer Cell.

[133] Zhou X., Thorgeirsson S.S., Popescu N.C. (2004). Restoration of DLC-1 gene expression induces apoptosis and inhibits both cell growth and tumorigenicity in human hepatocellular carcinoma cells. Oncogene.

[134] Ullmannova V., Popescu N.C. (2006). Expression profile of the tumor suppressor genes DLC-1 and DLC-2 in solid tumors. Int. J. Oncol..

[135] Syed V., Mukherjee K., Lyons-Weiler J., Lau K.M., Mashima T., Tsuruo T., Ho S.M. (2005). Identification of ATF-3, caveolin-1, DLC-1, and NM23-H2 as putative antitumorigenic, progesterone-regulated genes for ovarian cancer cells by gene profiling. Oncogene.

[136] Nagaraja G.M., Kandpal R.P. (2004). Chromosome 13q12 encoded Rho GTPase activating protein suppresses growth of breast carcinoma cells, and yeast two-hybrid screen shows its interaction with several proteins. Biochem. Biophys. Res. Commun..

[137] Leung T.H.Y., Ching Y.P., Yam J.W.P., Wong C.M., Yau T.O., Jin D.Y., Ng I.O.L. (2005). Deleted in liver cancer 2 (DLC2) suppresses cell transformation by means of inhibition of RhoA activity. Proc. Natl. Acad. Sci. USA.

[138] Bateman A. (2019). UniProt: A worldwide hub of protein knowledge. Nucleic Acids Res..

[139] Muhammed M.T., Aki-Yalcin E. (2019). Homology modeling in drug discovery: Overview, current applications, and future perspectives. Chem. Biol. Drug Des..

[140] Tuccinardi T., Manetti F., Schenone S., Martinelli A., Botta M. (2007). Construction and validation of a RET TK catalytic domain by homology modeling. J. Chem. Inf. Model..

[141] Lee J., Freddolino P.L., Zhang Y. (2017). Ab initio protein structure prediction. From Protein Structure to Function with Bioinformatics.

[142] Hulce J.J., Cognetta A.B., Niphakis M.J., Tully S.E., Cravatt B.F. (2013). Proteome-wide mapping of cholesterol-interacting proteins in mammalian cells. Nat. Methods.

[143] Tsujishita Y., Hurley J.H. (2000). Structure and lipid transport mechanism of a StAr-related domain. Nat. Struct. Biol..

[144] Horvath M.P., George E.W., Tran Q.T., Baumgardner K., Zharov G., Lee S., Sharifzadeh H., Shihab S., Mattinson T., Li B. (2016). Structure of the lutein-binding domain of human StARD3 at 1.74 Å resolution and model of a complex with lutein. Acta Crystallogr. Sect. Struct. Biol. Commun..

[145] RCSB PDB–3P0L: Human Steroidogenic Acute Regulatory Protein. https://www.rcsb.org/structure/3P0L.

[146] RCSB PDB–5I9J: Structure of the Cholesterol and Lutein-Binding Domain of Human STARD3 at 1.74A. https://www.rcsb.org/structure/5i9j.

[147] RCSB PDB–6L1D: Structure of Human StAR-related Lipid Transfer Protein 4. https://www.rcsb.org/structure/6L1D.

[148] RCSB PDB–2R55: Human StAR-Related Lipid Transfer Protein 5. https://www.rcsb.org/structure/2r55.

[149] RCSB PDB–2MOU: Solution Structure of StAR-related Lipid Transfer Domain Protein 6 (STARD6). https://www.rcsb.org/structure/2MOU.

[150] Altschul S.F., Madden T.L., Schäffer A.A., Zhang J., Zhang Z., Miller W., Lipman D.J. (1997). Gapped BLAST and PSI-BLAST: A new generation of protein database search programs. Nucleic Acids Res..

[151] Reitz J., Gehrig-Burger K., Strauss J.F., Gimpl G. (2008). Cholesterol interaction with the related steroidogenic acute regulatory lipid-transfer (START) domains of StAR (STARD1) and MLN64 (STARD3). FEBS J..

[152] Murcia M., Faráldo-Gómez J.D., Maxfield F.R., Roux B. (2006). Modeling the structure of the StART domains of MLN64 and StAR proteins in complex with cholesterol. J. Lipid Res..

[153] Kumar K.K., Devi B.U., Neeraja P. (2018). Molecular activities and ligand-binding specificities of StAR-related lipid transfer domains: Exploring integrated in silico methods and ensemble-docking approaches. SAR QSAR Environ. Res..

[154] Poli G., Martinelli A., Tuccinardi T. (2016). Reliability analysis and optimization of the consensus docking approach for the development of virtual screening studies. J. Enzyme Inhib. Med. Chem..

[155] Dal Piaz F., Vera Saltos M.B., Franceschelli S., Forte G., Marzocco S., Tuccinardi T., Poli G., Nejad Ebrahimi S., Hamburger M., De Tommasi N. (2016). Drug Affinity Responsive Target Stability (DARTS) Identifies Laurifolioside as a New Clathrin Heavy Chain Modulator. J. Nat. Prod..

[156] Zhang M., Liu P., Dwyer N.K., Christenson L.K., Fujimoto T., Martinez F., Comly M., Hanover J.A., Joan Blanchette-Mackie E., Strauss J.F. (2002). MLN64 mediates mobilization of lysosomal cholesterol to steroidogenic mitochondria. J. Biol. Chem..

[157] Watari H., Arakane F., Moog-Lutz C., Kallen C.B., Tomasetto C., Gerton G.L., Rio M.C., Baker M.E., Strauss J.F. (1997). MLN64 contains a domain with homology to the steroidogenic acute regulatory protein (StAR) that stimulates steroidogenesis. Proc. Natl. Acad. Sci. USA.

[158] Charman M., Kennedy B.E., Osborne N., Karten B. (2010). MLN64 mediates egress of cholesterol from endosomes to mitochondria in the absence of functional Niemann-Pick Type C1 protein. J. Lipid Res..

[159] Balboa E., Castro J., Pinochet M.J., Cancino G.I., Matías N., José Sáez P., Martínez A., Álvarez A.R., Garcia-Ruiz C., Fernandez-Checa J.C. (2017). MLN64 induces mitochondrial dysfunction associated with increased mitochondrial cholesterol content. Redox Biol..

[160] Montero J., Morales A., Llacuna L., Lluis J.M., Terrones O., Basañez G., Antonsson B., Prieto J., García-Ruiz C., Colell A. (2008). Mitochondrial cholesterol contributes to chemotherapy resistance in hepatocellular carcinoma. Cancer Res..

[161] Sahlberg K.K., Hongisto V., Edgren H., Mäkelä R., Hellström K., Due E.U., Moen Vollan H.K., Sahlberg N., Wolf M., Børresen-Dale A.L. (2013). The HER2 amplicon includes several genes required for the growth and survival of HER2 positive breast cancer cells. Mol. Oncol..

[162] Kao J., Pollack J.R. (2006). RNA interference-based functional dissection of the 17q12 amplicon in breast cancer reveals contribution of coamplified genes. Genes Chromosom. Cancer.

[163] Staaf J., Jönsson G., Ringnér M., Vallon-Christersson J., Grabau D., Arason A., Gunnarsson H., Agnarsson B.A., Malmström P.O., Johannsson O.T. (2010). High-resolution genomic and expression analyses of copy number alterations in HER2-amplified breast cancer. Breast Cancer Res..

[164] Qi F., Qin W.X., Zang Y.S. (2019). Molecular mechanism of triple-negative breast cancer-associated BRCA1 and the identification of signaling pathways. Oncol. Lett..

[165] Kim S.J. (2014). PPP1R1B-STARD3 chimeric fusion transcript in human gastric cancer promotes tumorigenesis through activation of PI3K/AKT signaling. Oncogene.

[166] Dong J.T. (2006). Prevalent mutations in prostate cancer. J. Cell. Biochem..

[167] Peretti D., Kim S.H., Tufi R., Lev S. (2020). Lipid Transfer Proteins and Membrane Contact Sites in Human Cancer. Front. Cell Dev. Biol..

[168] Alpy F., Rousseau A., Schwab Y., Legueux F., Stoll I., Wendling C., Spiegelhalter C., Kessler P., Mathelin C., Rio M.C. (2013). STARD3 or STARD3NL and VAP form a novel molecular tether between late endosomes and the ER. J. Cell Sci..

[169] Di Mattia T., Martinet A., Ikhlef S., McEwen A.G., Nominé Y., Wendling C., Poussin-Courmontagne P., Voilquin L., Eberling P., Ruffenach F. (2020). FFAT motif phosphorylation controls formation and lipid transfer function of inter-organelle contacts. EMBO J..

[170] Amarilio R., Ramachandran S., Sabanay H., Lev S. (2005). Differential regulation of endoplasmic reticulum structure through VAP-Nir protein interaction. J. Biol. Chem..

[171] Loewen C.J.R., Levine T.P. (2005). A highly conserved binding site in vesicle-associated membrane protein-associated protein (VAP) for the FFAT motif of lipid-binding proteins. J. Biol. Chem..

[172] Kaiser S.E., Brickner J.H., Reilein A.R., Fenn T.D., Walter P., Brunger A.T. (2005). Structural basis of FFAT motif-mediated ER targeting. Structure.

[173] Hayashi T., Rizzuto R., Hajnoczky G., Su T.P. (2009). MAM: More than just a housekeeper. Trends Cell Biol..

[174] Nara A. (2015). STARD3/MLN64 is Striving at Membrane Contact Sites: Intracellular Cholesterol Trafficking for Steroidogenesis in Human Placental Cells. Am. J. Life Sci..

[175] Di Mattia T., Wilhelm L.P., Ikhlef S., Wendling C., Spehner D., Nominé Y., Giordano F., Mathelin C., Drin G., Tomasetto C. (2018). Identification of MOSPD2, a novel scaffold for endoplasmic reticulum membrane contact sites. EMBO Rep..

[176] Höglinger D., Burgoyne T., Sanchez-Heras E., Hartwig P., Colaco A., Newton J., Futter C.E., Spiegel S., Platt F.M., Eden E.R. (2019). NPC1 regulates ER contacts with endocytic organelles to mediate cholesterol egress. Nat. Commun..

[177] Forbes S.A., Beare D., Boutselakis H., Bamford S., Bindal N., Tate J., Cole C.G., Ward S., Dawson E., Ponting L. (2017). COSMIC: Somatic cancer genetics at high-resolution. Nucleic Acids Res..

[178] Wolber G., Langer T. (2005). LigandScout: 3-D pharmacophores derived from protein-bound ligands and their use as virtual screening filters. J. Chem. Inf. Model..

[179] Chitrala K.N., Yeguvapalli S. (2014). Ligand-based virtual screening to predict inhibitors against metastatic lymph node 64. J. Recept. Signal. Transduct..

