# Supplementary material for: STARD3: A Prospective Target for Cancer Therapy
Source: Cancers (Basel). 2021 Sep 18;13(18):4693. doi: 10.3390/cancers13184693 (PMC8472075; doi:10.3390/cancers13184693)
Supplement: Supplementary file 1 [file cancers-13-04693-s001.zip › cancers-1375693-supplementary.pdf]

Table supplementary 1. Predicted pathogenic mutations in STARD3

Primary Tissue	AA Mutation	FATHMM score	CDS Mutation	Histology	Pubmed Id	Somatic Status
Breast	p.S25*	0.96	c.74C>G	Carcinoma	-	Confirmed Somatic
Breast	p.S47F	0.97	c.140C>T	Carcinoma	-	Confirmed Somatic
Breast	p.I64=	0.88	c.192C>T	Carcinoma	-	Confirmed Somatic
Breast	p.V189I	0.97	c.565G>A	Carcinoma	-	Confirmed Somatic
Breast	p.E223K	0.99	c.667G>A	Carcinoma	-	Confirmed Somatic
Breast	p.R351Q	0.99	c.1052G>A	Carcinoma	-	Confirmed Somatic
Adrenal gland	p.A187S	0.99	c.559G>T	Adrenal cortical carcinoma	24747642	Confirmed Somatic
Biliary tract	p.L309=	0.95	c.927G>A	Carcinoma	-	Confirmed Somatic
Central nervous system and endometrium	p.R356C	0.98	c.1066C>T	Glioma	28263318	Confirmed Somatic on two patient samples
Cervix	p.E220K	0.99	c.658G>A	Carcinoma	-	Confirmed Somatic on two patient samples
Cervix	p.V390M	0.99	c.1168G>A	Carcinoma	-	Confirmed Somatic
Endometrium	p.E71K	0.99	c.211G>A	Carcinoma	-	Confirmed Somatic
Haematopoietic and lymphoid	p.K42E	0.81	c.124A>G	Lymphoid neoplasm	23415222	Confirmed Somatic
Kidney	p.N81K	0.98	c.243C>A	Carcinoma	29088767	Confirmed Somatic
Kidney	p.?	0.99	c.795+1G>A	Carcinoma	-	Confirmed Somatic
Kidney	p.K377I	0.98	c.1130A>T	Carcinoma	29118224	Confirmed Somatic on two patient samples
Kidney and prostate	p.R437H	0.98	c.1310G>A	Carcinoma	25401301; 26000489	Confirmed Somatic in two patient samples
Large intestine and stomach	p.R117Q	0.92	c.350G>A	Carcinoma	24599305	Confirmed Somatic on five samples (3 large intesine and 2 stomach carcinoma
Large intestine	p.G148W	0.99	c.442G>T	Carcinoma	-	Confirmed Somatic
Large intestine		0.98	c.734C>A	Carcinoma	26109429	Confirmed Somatic in two patient samples
Large intestine	p.E257D	0.90	c.771G>T	Carcinoma	-	Confirmed Somatic

Large intestine	p.V271M	0.99	c.811G>A	Carcinoma	-	Confirmed Somatic

Primary Tissue	AA Mutation	FATHMM score	CDS Mutation	Histology	Pubmed Id	Somatic Status
Liver	p.G23V	0.93	c.68G>T	Other	-	Confirmed Somatic
Liver	p.F96L	0.99	c.286T>C	Other	-	Confirmed Somatic in two patient samples
Liver	p.?	0.97	c.298-1G>T	Other	-	Confirmed Somatic
Liver	p.G216A	0.99	c.647G>C	Carcinoma	-	Confirmed Somatic
Liver	p.A247S	0.89	c.739G>T	Other	-	Confirmed Somatic
Liver	p.L302=	0.91	c.906G>A	Other	-	Confirmed Somatic
Liver	p.L361F	0.86	c.1083G>T	Other	-	Confirmed Somatic
Lung	p.S25L	0.95	c.74C>T	Carcinoma	27923066	Confirmed Somatic
Lung	p.R43L	0.93	c.128G>T	Carcinoma	-	Confirmed Somatic
Lung	p.F53=	0.97	c.159C>T	Carcinoma	27923066	Confirmed Somatic
Lung	p.V57=	0.82	c.171C>T	Carcinoma	27923066	Confirmed Somatic
Lung and NS	p.E85K	1	c.253G>A	Carcinoma	24265153	confirmed somatic on two patient samples
Lung	p.R119Q	0.87	c.356G>A	Carcinoma	-	Confirmed Somatic
Lung and stomach	p.A162T	0.99	c.484G>A	Carcinoma	22941188	confirmed somatic on two patient samples
Lung	p.Q321L	0,98	c.962A>T	Carcinoma	-	Confirmed Somatic
Lung	p.V333M	0.95	c.997G>A	Carcinoma	-	Confirmed Somatic
Lung	p.G386=	0.76	c.1158G>T	Carcinoma	-	Confirmed Somatic
Lung	p.R437S	0.92	c.1309C>A	Carcinoma	26168399	Confirmed Somatic
NS	p.R380Q	1	c.1139G>A	Malignant melanoma	24265153	Confirmed Somatic
Oesophagus	p.R345G	0.96	c.1033A>G	Carcinoma	25151357	Confirmed Somatic
Ovary	p.R413L	0.97	c.1238G>T	Carcinoma	-	Confirmed Somatic
Pancreas	p.T128M	0.98	c.383C>T	Carcinoma	25743105	Confirmed Somatic
Pancreas	p.?	0.95	c.547+3G>C	Carcinoma	-	Confirmed Somatic
Pancreas	p.W310*	0.99	c.930G>A	Carcinoma	-	Confirmed Somatic

Primary Tissue	AA Mutation	FATHMM score	CDS Mutation	Histology	Pubmed Id	Somatic Status
Pancreas	p.E429Q	0.97	c.1285G>C	Carcinoma	23103869	Confirmed Somatic on two patient samples
Pancreas	p.E440K	0.99	c.1318G>A	Carcinoma	-	Confirmed Somatic
Prostate	p.A114V	0.97	c.341C>T	Carcinoma	-	Confirmed Somatic
Skin	p.E13Q	0.98	c.37G>C	Carcinoma	24662767	Confirmed Somatic
Skin	p.V49I	0.98	c.145G>A	Carcinoma	24662767	Confirmed Somatic
Skin	p.L66M	0.96	c.196C>A	Malignant melanoma	-	Confirmed Somatic
Skin	p.V100=	0.82	c.300C>T	Carcinoma	24662767	Confirmed Somatic
Skin	p.R105C	0.96	c.313C>T	Carcinoma	25303977	Confirmed Somatic
Skin	p.E257=	0.85	c.771G>A	Carcinoma	24662767	Confirmed Somatic
Skin	p.P290L	0.98	c.869C>T	Carcinoma	25759019	Confirmed Somatic
Skin	p.I319=	0.73	c.957C>T	Carcinoma	25303977	Confirmed Somatic
Skin	p.S343F	0.89	c.1028C>T	Carcinoma	25759019	Confirmed Somatic
Skin	p.S343=	0.79	c.1029C>T	Carcinoma	25303977	Confirmed Somatic
Skin	p.G386E	0.99	c.1157G>A	Malignant melanoma	26214590	Confirmed Somatic
Skin	p.I389=	0.75	c.1167C>T	Malignant melanoma	-	Confirmed Somatic on two patient samples
Skin	p.P397H	0.99	c.1190C>A	Malignant melanoma	-	Confirmed Somatic
Skin and pancreas	p.E440K	0.99	c.1318G>A	Carcinoma	25303977	Confirmed Somatic on two patient samples
Small intestine	p.R182Q	0.98	c.545G>A	Adenoma	26806338	Confirmed Somatic
Soft tissue	p.S209L	0.99	c.626C>T	Liposarcoma	26643872	Confirmed Somatic
Stomach	p.A18V	0.92	c.53C>T	Carcinoma	22037554;24816253	Confirmed somatic on two patient samples
Stomach	p.S142Y	0.98	c.425C>A	Carcinoma	24816253	Confirmed Somatic
Stomach	p.R193C	0.94	c.577C>T	Carcinoma	-	Confirmed Somatic
Stomach	p.R240C	0.93	c.718C>T	Carcinoma	25042771	Confirmed Somatic
Stomach	p.S393*	0.92	c.1178C>A	Carcinoma	-	Confirmed Somatic
Urinary tract	p.D60N	0.99	c.178G>A	Carcinoma	24121792	Confirmed Somatic
Upper aerodigestive tract	p.I301F	0.98	c.901A>T	Carcinoma	-	Confirmed Somatic

* : Non sense mutation.
= : silent mutation
?: Unknown mutation. No information available
FATHMM: Functional Analysis through Hidden Markov Models. Scores ≥ 0.7 indicate pathogenic mutations.
Confirmed somatic: tumour and germ-line samples of the same patient have been analysed. A variant allele is present only in tumour sample.
Previously reported: no germ-line allele information available, but the variant has been reported 'Confirmed Somatic' in a normal-tumour sample pair from another patient.
